# Crosstalk between Autophagy and Apoptosis: Potential and Emerging Therapeutic Targets for Cardiac Diseases

**DOI:** 10.3390/ijms17030332

**Published:** 2016-03-03

**Authors:** Meng Li, Ping Gao, Junping Zhang

**Affiliations:** 1 Graduate School, Tianjin University of Traditional Chinese Medicine, Tianjin 300193, China; zhongyilimeng@163.com; 2 Department of Medical Imaging, Urumqi General Hospital of Lanzhou Military Area Command, Urumqi 830000, China; doctorgaoping@sina.com; 3 Department of cardiology, First Teaching Hospital of Tianjin University of Traditional Chinese Medcine, Tianjin 300192, China

**Keywords:** autophagy, apoptosis, crosstalk, cardiovascular diseases

## Abstract

Autophagy is a cell survival process which is related to breaking down and reusing cytoplasm components. Moreover, autophagy regulates cell death under certain conditions. Apoptosis has the characteristics of chromatin agglutination and the shrinking of nuclear and apoptosis body form. Even if the mechanisms of autophagy and apoptosis have differences, some proteins modulate both autophagy and apoptosis. Crosstalk between them exists. This review highlights recent advances in the interaction of autophagy and apoptosis and its importance in the development of cardiovascular diseases.

## 1. Molecular Mechanisms of Autophagy

Autophagy is a complex and evolutionarily conserved process. It is involved in the degrading of abnormal proteins and organelles [1]. Autophagy is significant for maintaining cellular homeostasis under regular conditions, and is rapidly triggered by different stimuli such as nutrient starvation [2], hypoxia [3], oxidative stress [4], pathogen infection [5] and endoplasmic reticulum stress [6]. There are mainly three kinds of autophagy: (1) microautophagy, which directly sequesters and engulfs the cytoplasmic constituents via indentation inwards of the lysosome membrane [7]; (2) chaperone-controlled autophagy, where cytosolic proteins with the KFERQ-like motif are recognized by chaperones, then unfold and translocate into the lysosome through the lysosomal-associated membrane protein type 2A [8]; and (3) macroautophagy, which is characterized by formation of the autophagosome (a double-membrane sequestering compartment) and fusing with the lysosome to deliver the cytoplasmic cargo. The process of macroautophagy (hereinafter referred to as autophagy) is as follows: induction, nucleation of the autophagosome precursor (phagophore), phagophore expansion and autophagosome maturation, fusing with the lysosome, and recycling of the degraded cargo [9,10].

Autophagy Induction: The mammalian target of rapamycin (mTOR) integrates nutrient signals and cytokines from different pathways to inhibit autophagy and promote cell growth [11]. Under stress or nutrient starvation conditions, mTOR is inhibited, which initiates autophagy by formation of the Unc-51-Like Kinase (ULK) complex including ULK, Autophagy-related Protein 13 (Atg13) and FAK-family Interacting Protein of 200 kDa (FIP200) [12,13]. Then the ULK complex phosphorylates Activating Molecule in Beclin-1-Regulated Autophagy (AMBRA1), which activates the phosphatidylinositol-3-kinase (PI3K) complex composed of Vacuolar Protein Sorting 15 (VPS15), VPS34, Beclin-1 and AMBRA1 [14,15]. During autophagy initiation, Beclin-1 is phosphorylated. Then Beclin-1 is separated from the dynein motor complexes, which are positively regulated by AMBRA1 [16].

Autophagosome Formation: Once autophagy is induced, phagophore assembling is initiated by membrane nucleation [17]. The membranes mostly originate from the mitochondria [18], endoplasmic reticulum [19], trans-Golgi network [20], late endosomes [21], and plasma membrane [22]. Elongation and expansion of the phagophore membrane is an important stage in autophagosome formation. It is modulated by two inter-related systems of Atg12-Atg5-Atg16 and Atg8 [23]. In Atg12-Atg5-Atg16, Atg12 is initially triggered by the Atg7 in an ATP-dependent way. Then Atg12 is transferred to the E2-like conjugating enzyme Atg10 and forms the Atg12-Atg10 intermediate. Finally, Atg12 is covalently attached to Atg5. Further interplay between Atg5-Atg12 and the Atg16 homodimer leads to formation of the Atg12-Atg5-Atg16 complex. This complex locating to the phagophore is essential for autophagy. The second ubiquitin-like system induces the conjugation of phosphatidylethanolamine to Atg8/microtubule-associated protein 1 light chain 3 (LC3), which is subsequently processed by Atg4, Atg7 and Atg3 [24,25]. LC3-I is transformed into LC3-II. LC3-II is a special marker for the autophagosome [26].

**Figure 1:**
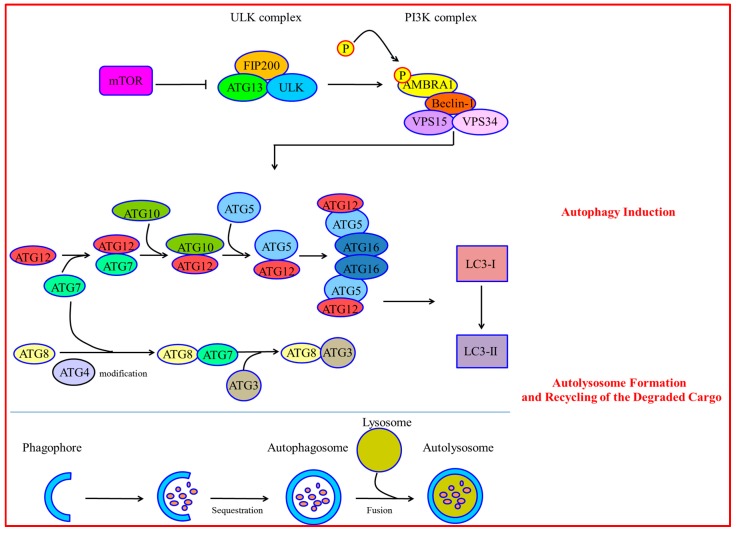
Autophagy Induction: Under stress or nutrient starvation conditions, mTOR is inhibited, which initiates autophagy by formation of the ULK complex including ULK, Atg13 and FIP200. Then the ULK complex phosphorylates AMBRA1, which activates the PI3K complex composed of VPS15, VPS34, Beclin-1 and AMBRA1. During autophagy initiation, Beclin-1 is phosphorylated and released from the dynein motor complex, which is positively regulated by AMBRA1. Autophagosome Formation: Elongation and expansion of the phagophore membrane is regulated by the two inter-related ubiquitin-like conjugation systems of Atg12-Atg5-Atg16 and Atg8. In the Atg12-Atg5-Atg16 system, Atg12 is initially activated by the E1-like activating enzyme Atg7 in an ATP-dependent way; then Atg12 is transferred to the E2-like conjugating enzyme Atg10 and forms the Atg12-Atg10 intermediate; finally, Atg12 is covalently attached to Atg5. Further interaction between the Atg5-Atg12 heterodimer and Atg16 homodimer leads to the formation of the Atg12-Atg5-Atg16 complex. The second ubiquitin-like system induces the conjugation of phosphatidylethanolamine to Atg8/microtubule-associated protein 1 light chain 3 (LC3), which is subsequently processed by Atg4, Atg7 and Atg3. LC3-I is transformed into LC3-II. Autolysosome Formation and Recycling of the Degraded Cargo: autolysosome formation originates from the delivery and fusion of the autophagosome to lysosome.

Autolysosome Formation and Recycling of the Degraded Cargo: Autolysosome formation originates from the transmitting and fusion of the autophagosome to lysosome. It is regulated by cytoskeleton and lysosomal membrane proteins [27]. LAMP1/2 regulates autophagosome maturation. Gene mutation of LAMP2 causes Danon disease. Autophagosome accumulation and cardiomyocyte hypertrophy are characteristics of Danon disease. [28,29]. Once the autolysosome forms, the inner cargoes are degraded by lysosomal hydrolases. Catabolic products such as amino acids release into the cytoplasm for recycling and can be used as new resources [30] (Figure 1).

## 2. Molecular Mechanisms of Apoptosis

Apoptosis is a process characterized by chromatin condensation, nuclear shrinkage and apoptosis body production [31]. The apoptotic signaling cascade mainly includes two pathways, intrinsic and extrinsic, and it gets triggered by different mitochondrial stimuli or by molecules binding to the cell-membrane receptor [32,33]. The intrinsic apoptosis signaling is induced by various stimuli, such as hypoxia [34], DNA damage [35], oxidative stress [36] and deprivation of growth factor [37]. It leads to mitochondrial membrane permeabilization. The integrity of mitochondria is regulated by different Bcl-2 superfamily members. They have the feature of the BH3 (Bcl-2 Homology) domain. Bcl-2 proteins are divided into two subcategories: pro-apoptotic and anti-apoptotic. Pro-apoptotic family members contain Bak, Bax, Bid, Bad, Noxa and PUMA. The anti-apoptotic family members include Bcl-2, Bcl-X_L_, Mcl-1, Bcl-W and A1/Bfl-1 [38]. The multi-domain pro-apoptotic proteins Bax and Bak are essential for inducing apoptosis. In response to stimulation of apoptosis, these proteins undergo conformational changes. It leads to their oligomerization on the outer membrane of mitochondria [39]. Bcl-2 proteins block this pathway by interacting with Bax and Bak. It inhibits mitochondria permeabilization and cell death [40]. After mitochondrial permeabilization, cytochrome c is released into the cytoplasm. Then cytochrome c binds to apoptotic protease-activating factor-1 (Apaf-1). It induces the conformational change and oligomerization of Apaf-1. This leads to the forming of a caspase-activating platform called the apoptosome. The apoptosome is comprised of Apaf-1, caspase-9 and cytochrome c. The apoptosome recruits, dimerizes and triggers caspase-9. Successively, it cleaves and induces caspase-3 and caspase-7 [41]. The last step of apoptosis is degrading DNA. The process is regulated by Endonuclease G. Endonuclease G is translocated from mitochondria to the nucleus and cleaves DNA [42].

**Figure 2:**
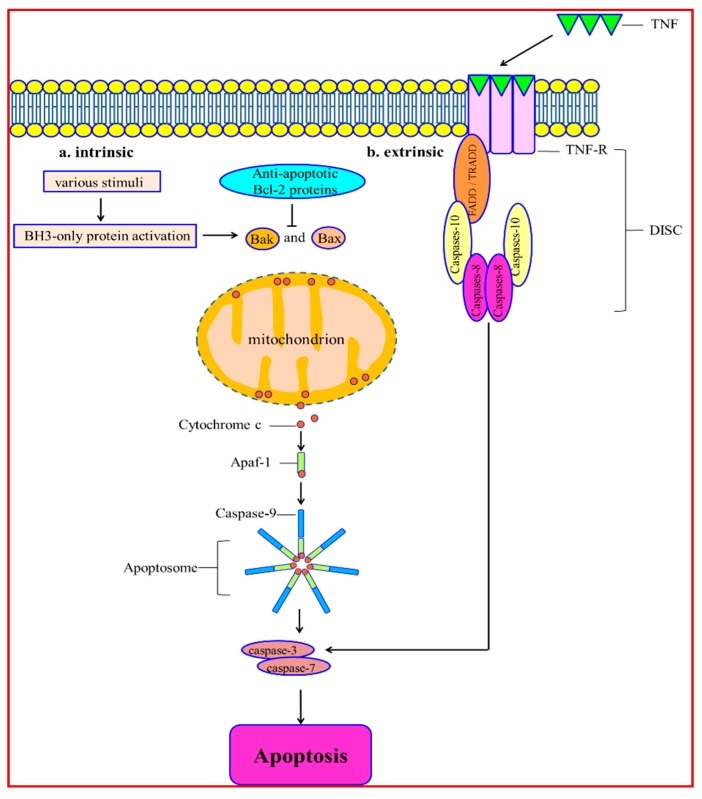
The intrinsic apoptotic pathway is triggered by various stimuli inducing mitochondrial membrane permeabilization. In response to apoptotic stimuli, these proteins undergo conformational changes, which lead to their oligomerization on the outer mitochondrial membrane. Anti-apoptotic Bcl-2 proteins block this process by interacting with Bax and Bak, which inhibits mitochondria permeabilization and subsequent cell death. After mitochondrial permeabilization, cytochrome c released into the cytosol binds to Apaf-1. It induces the conformational change and oligomerization of Apaf-1. This promotes apoptosome formation. The apoptosome is composed of Apaf-1, caspase-9 and cytochrome c. The apoptosome can recruit, dimerize and induce caspase-9. Successively, it leads to cleaving and inducing of caspase-3 and caspase-7. The last step of apoptosis is DNA degradation. The extrinsic apoptosis signal is triggered by activation of death receptors. The cognate extracellular death ligands refer to soluble molecules of the TNF family. They are released as homotrimers and bind to the TNF-R family. Ligand-binding makes the cell membrane receptors trimerize and activate. TNF-Rs have a death domain (DD) that can recruit other DD-containing proteins. These proteins include Fas-associated protein with death domain (FADD) and TNF-R type 1-associated death domain protein (TRADD). These proteins bind to caspase-8 and caspase-10, and then activate DISC. DISC primarily activates caspase-8 and promotes the cell death outcome. After the activation of caspase-8 and -10, caspase-3 and -7 are cleaved, which causes cell degradation.

The extrinsic apoptosis pathway is initiated through activating the death receptors. Death receptors bind to ligands and deliver apoptosis signaling. The cognate extracellular death ligands refer to soluble molecules of tumor necrosis factor (TNF). They are released as homotrimers and bind to the TNF-receptor (TNF-R). The TNF-R family is comprised of Fas/CD95, TNF-R1, TRAIL receptors-1 (TRAIL-R1), TRAIL-R2, DR-3 and DR-6. Ligand-binding makes the cell membrane receptors trimerize and activate [43]. TNF-Rs have a death domain (DD) that can recruit other DD-associated proteins. The DD-associated proteins include the Fas-associated protein with death domain (FADD) and TNF-R type 1-associated death domain protein (TRADD) [44,45]. These proteins bind to caspase-8 and -10. Then the death-inducing signaling complex (DISC) is activated. DISC primarily activates caspase-8 and promotes the cell death outcome. Caspase-3 and -7 are cleaved after induction of caspase-8 and -10, which causes cell degradation [46,47] (Figure 2).

## 3. Crosstalk between Autophagy and Apoptosis

Emerging evidence suggests interactions among the crucial proteins of autophagy and apoptosis, which underlie the molecular mechanism of the crosstalk between them. The essential factors connecting autophagy and apoptosis are depicted in Table 1.

### 3.1. Bcl-2/Beclin-1

The B-cell lymphoma 2 (Bcl-2) family proteins inhibit cytochrome c releasing from the mitochondria. It plays a critical role in the apoptosis process [48]. Beclin-1 is a component of the class III PI3K/Vps34 complex and is necessary for forming the autophagy vesicle [49]. Bcl-2 binds to Beclin-1 and segregates Beclin-1 away from class III PI3K, leading to an inhibition of autophagic response [50]. In contrast, mutations of either Beclin-1’s domain or the BH3 receptor domain within Bcl-2 will disrupt the Bcl-2-Beclin-1 complex, which promotes autophagic activity [51,52]. The interplay between Bcl-2 and Beclin-1 is essential to regulate the crosstalk between autophagy and apoptosis [53,54].

Under the condition of sufficient nutrition, Beclin-1 and Bax/Bak bind to Bcl-2 or Bcl-X_L_. It inhibits activation of autophagy and apoptosis, respectively [55,56]. Under conditions of starvation, autophagy is essential to guarantee cell survival. C-Jun N-terminal protein kinase 1 (JNK1) is activated and phosphorylates various residuum (Thr69, Ser70, and Ser87) of Bcl-2’s regulatory loop. It leads to Bcl-2-Beclin-1 complex destruction, which induces autophagy [57]. After autophagy activation, death-associated protein kinase (DAPK) phosphorylates the Thr119 of Beclin-1. It induces Beclin-1 separating from Bcl-2 [58]. Moreover, phosphorylating Beclin-1 on Thr119 decreases the Bcl-X_L_-Beclin-1 combining. It promotes autophagosome formation [59]. The cytosolic translocation of high mobility group box 1 (HMGB1) is another factor promoting Bcl-2/Bcl-X_L_ separation from Beclin-1. The intramolecular disulphide bridge (C23/45) of HMGB1 interacts with Beclin-1, which causes HMGB-1 to replace Bcl-2 [60,61]. Undergoing a long period of starvation cannot be relieved by autophagy. Phosphorylated Bcl-2 combines with Bax and inhibits apoptosis. The phosphorylated Bcl-2 protects cells against apoptosis through preserving the mitochondrial membrane completeness and preventing the pro-apoptosis proteins from releasing into the cytoplasm. However, in the situation of extreme starvation, JNK1 promotes hyper-phosphorylation of Bcl-2. It promotes Bcl-2 separating from Bax. Then it induces apoptosis via caspase-3-dependent pathways and, subsequently, a safe cell death [62].

### 3.2. Atgs

Autophagy-related proteins (Atgs) involved in various stages of autophagy have also been shown to regulate the apoptotic pathway [85]. Gene mutation or inhibition of these specific Atgs may affect the apoptosis process [86]. Atg3 is a non-canonical ubiquitination E2 enzyme regulating the conjugation of ubiquitin-like Atg8 to phosphatidylethanolamine in the autophagy process [25]. In addition, recent studies have shown that Atg12 covalently conjugates to Atg3. The Atg3-Atg12 complex localizes to the mitochondrial outer membrane, regulating mitochondrial homeostasis and cell death. Atg12 conjugation to Atg3 sensitizes cells to mitochondria-mediated apoptosis. However, it has no effect on death receptor–mediated apoptosis [87]. Disturbing the complex formation, selective mitochondrial autophagy (also called mitophagy) is reduced significantly, but it has no effect upon non-selective autophagy [88].

Atg4 is a cysteine protease cleaving the C-terminus of LC3, which has an effect on the covalent attachment of newly synthesized Atg8 to PE and on the delipidation of Atg8 at the lysosomal fusion stage [64]. Atg4D, a human Atg4 member, is cleaved by caspase3 and generates two fragments in the apoptosis cell. The N-terminal fragment of Atg4D cleaves and delipidates the Atg8 paralogue γ-aminobutyric acid receptor-associated protein-like 1 (GABARAP-L1), which leads to the decrease of autophagosome formation. The C-terminal fragment with a putative BH3 domain is recruited to the mitochondrial matrix, promoting the mitochondria-mediated apoptosis [63,89].

Covalent conjugation of the autophagy-related proteins Atg5 and Atg12 involved in an ubiquitylation-like process is essential to autophagosome formation. Atg5 and Atg12 are, therefore, integral parts of the autophagic machinery and are required for the induction of autophagy [65]. Hence, Atg5 and Atg12 are absolutely necessary for autophagic activity. Interestingly, it has been found that Atg5 and Atg12 also participate in apoptosis initiation in response to various stress signals. Moreover, non-conjugated forms of Atg5 and Atg12 have an effect on the induction of apoptosis, which indicates that their effect on apoptosis is likely to be independent of their effect on autophagy. Atg5 has a double role in regulating autophagy and apoptosis. One study reported that over-expression of Atg5 made the tumor cells sensitive to chemotherapy, while silencing the Atg5 gene with short interfering RNA made the cells partially resistant to chemotherapy. This study showed that, during apoptosis, Atg5 was cleaved by calpains, producing an amino-terminal cleavage product. Truncated Atg5 moved from the cytoplasm to mitochondria. Then it interacted with Bcl-X_L_ and promoted cytochrome c release and caspase activation [66]. Atg5 cleavage was found independent of the apoptotic stimulus and cell type. It was indicated that calpain induction and Atg5 cleavage were universal phenomena in apoptotic cells. On the contrary, without Atg5 in mitochondria, autophagy takes place [67].

Atg12 has a dual function, participating in autophagy and apoptosis, which connects both of the processes. Non-conjugated Atg12 can combine with and inhibit Mcl-1 and Bcl-2 by a BH3-like motif, which positively regulates mitochondrial apoptosis. In the apoptosis cell, knockout of Atg12 inhibits Bax induction and cytochrome c release. On the contrary, aberrant expression of Atg12 represses the anti-apoptotic activity of Mcl-1 [90]. In addition, a recent study demonstrated that free Atg12 was unstable. It could be broken down in a proteasome-dependent way. Atg12 could be directly ubiquitinated, which promotes the proteasomal degradation. Free Atg12 could cause proteasome inhibitor–regulated apoptosis, indicating proteasome inhibitors might be potential anticancer agents in clinical practice [68].

### 3.3. Caspases

Caspases are both the initiators and effectors participating in apoptotic cascades [69]. Recently, it has been found that caspases participate in regulating the crosstalk between autophagy and apoptosis [91]. Various pro-apoptosis pathways can induce caspases to trigger apoptosis. Moreover, activated caspases could cleave and break down the critical autophagic proteins (such as Beclin-1, p62, Atg3, Atg4D, Atg5, Atg7, and AMBRA1). It leads to an inactivation of their autophagic function [73,75,76,92,93,94]. Surprisingly, some pro-autophagic proteins can even be transformed into pro-apoptotic proteins to initiate apoptosis cell death after being cleaved by caspases. In addition, autophagy can have an effect on apoptotic cascades through modulating the caspases [94].

Caspase-2 is an important regulator of cascades in a context-dependent way [77]. Recent research reported that mice neurons in the absence of caspase-2 cannot execute apoptosis, while autophagy is activated at an early stage. It causes a response to rotenone-regulated mitochondrial oxidative stress [95]. It has also been found that, in mouse embryonic fibroblasts, a lack of caspase-2 contributes to an enhanced autophagy [96].

Caspase-3 is a predominant effector caspase in apoptosis [70]. However, accumulating studies have shown that caspase-3 is essential to autophagic activity. A study reported that, during staurosporine-induced apoptosis, caspase-3 could cleave Beclin-1 on 124 and 149. It inhibited autophagy and activated apoptosis in HeLa cells [71]. Another study found that caspase-3, together with other caspases, cleaved Beclin-1 in the apoptosis process. It regulated by IL-3 deprivation in culture medium, blocking autophagic activity and promoting the pro-apoptotic stimulus. The Beclin-1 C-terminal fragment localized at the mitochondria. Subsequently, it sensitized the cell to apoptosis [92].

Caspase-6 is also an effector caspase in apoptosis [97]. It has been demonstrated that caspase-6 cleaves p62 and Atg3, which suggests its importance in mediating autophagy [72]. Moreover, when melanoma cell lines suffer arginine withdrawal, TRAIL-induced caspase-6 activation disrupts autophagy by cleavage of two crucial autophagy proteins, Atg5 and Beclin-1 [76].

Caspase-8 is an essential trigger involved in death receptor–induced apoptosis [98]. The increasing evidence indicates that caspase-8 also participates in regulating autophagy. During the death receptor–triggered apoptosis, caspase-8 cleaves Atg3, targeting the conserved LETD sequence (Atg3 amino acids 166–169), which inactivates the pro-autophagic activity. In addition, caspase-8 could prevent T cells from hyperactive autophagy [93].

Caspase-9 is also a key triggering caspase participating in intrinsic apoptosis [74]. It has been reported that caspase-9 interacts with Atg7 at the C-terminal region. It promotes LC3-II formation and autophagy activity. The interplay between caspase-9 and Atg7 hinders the recruiting and processing of caspase-9 in apoptosomes, inhibiting caspase-9 activation and apoptosis [94]. Moreover, in breast cancer MCF-7 cells, suppression of caspase-9 can block the autophagic flux and induce the cell death by inhibiting cytoprotective autophagy [99].

### 3.4. p53

p53, a signal transduction integrator, can be induced by diverse abnormal conditions, including hypoxia [100], DNA damage [78], nutrient stress [101], and ischemia-reperfusion [102]. p53 has an effect on regulating apoptosis both through the intrinsic and extrinsic pathways. In the nucleus, p53 promotes the pro-apoptotic proteins (such as Bax, Bid, PUMA, and Noxa) and inhibits Bcl-2 expression, which triggers the intrinsic apoptotic pathway. In the cytoplasm, p53 promotes the TRAIL receptor and Fas receptor, causing the initiation of the extrinsic apoptotic pathway [103]. In addition, p53 can activate Apaf-1 of the apoptosome [104]. Recently, an increasing number of studies have indicated that p53 is also involved in the regulation of autophagy. It is reported that genotoxic stress induces autophagy through transcriptional activation of a direct p53 target gene, damage-regulated autophagy modulator (DRAM), whose signaling cascade promotes autolysosome formation. DRAM is essential for the network regulating p53-regulated apoptosis and autophagy [79]. Another study showed cytoplasmic p53 suppressed autophagy by inactivating AMP activated protein kinase (AMPK) and subsequently activating mTOR signaling [80]. It also has been investigated that, under the nutrient deprivation condition, p53 post-transcriptionally downregulates LC3, which controls the autophagic flux and prevents the cells from ‘‘autophagic burst’’ [82]. Moreover, inhibition of p53 triggers autophagy mostly in the G_1_ phase and less in S phase, but never in the G_2_/M phases. It is strictly cell cycle–dependent [81].

### 3.5. FLIP

**Figure 3:**
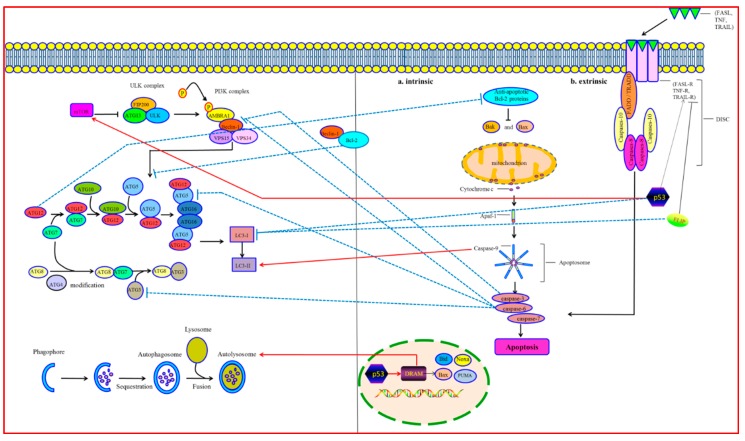
Crosstalk between Autophagy and Apoptosis. **Bcl-2/Beclin-1:** The interaction between the anti-apoptotic protein Bcl-2 and the autophagy protein Beclin-1 is essential to regulate the switch between autophagy and apoptosis. Bcl-2 binds to Beclin-1 and segregates Beclin-1 away from class III PI3K, leading to an inhibition of autophagic response. **Atgs:** Atg12 has a dual function of participating in autophagy and apoptosis, which connects both of the processes. Non-conjugated Atg12 can bind to Bcl-2 through a unique BH3-like motif, which positively regulates mitochondrial apoptosis. **Caspases:** caspases, a family of cysteine proteases, are both the initiators and effectors participating in apoptotic cascades. Caspase-3 can cleave Beclin-1 and inhibit autophagy. Caspase-6 can cleave Atg3, Atg5 and Beclin-1, which regulates autophagy. Caspase-9 can promote the Atg7-dependent formation of autophagosomal LC3-II and autophagic activity. **p53:** In nucleus, p53 promotes the expression of pro-apoptotic proteins (such as Bax, Bid, PUMA, and Noxa), which triggers the intrinsic apoptotic pathway; in cytoplasm, p53 increases the expression of TRAIL receptor and Fas receptor, causing the initiation of the extrinsic apoptotic pathway. p53 is also involved in the regulation of autophagy. Autophagy can be induced by transcriptional activation of DRAM, promoting autolysosome formation. DRAM seems to be a critical component of the network regulating p53-mediated apoptosis and autophagy. Cytoplasmic p53 suppresses autophagy by activating mTOR signaling. Under the nutrient deprivation condition, p53 post-transcriptionally downregulates LC3, which controls the autophagic flux and prevents the cells from ‘‘autophagic burst’’. **FLIP:** FLIP is an anti-apoptotic protein, suppressing the death receptor–mediated apoptosis. FLIP competes with LC3 for binding of Atg3 and inhibits LC3 lipidation, which suppresses autophagy. Red arrows indicate stimulatory inputs. Blue bars indicate inhibitory interactions. For clarity, some of the signaling connections between autophagy and apoptosis are not shown. See text for details.

FADD-like IL-1β-converting enzyme-inhibitory protein (FLIP) is an anti-apoptotic protein, suppressing death receptor–mediated apoptosis [83]. Recently, it has been shown that FLIP competes with LC3 for the binding of Atg3 and inhibits LC3 lipidation, which suppresses autophagy. On the contrary, once the autophagy is triggered, the interaction of FLIP and Atg3 is significantly decreased [105] (Figure 3).

### 3.6. Mitoptosis

Mitoptosis is an apoptotic-like process inside mitochondria. It occurs mainly as an outcome of mitochondrial outer membrane permeabilization (MOMP) and potential loss. It has been demonstrated that dysfunction of the mitochondria and production of ROS are essential for inducing mitoptosis [106]. It also has been reported that following the Bax/Bak-regulated MOMP, DDP/TIMM8a, a mitochondrial intermembrane space (IMS) protein, is released into the cytoplasm where it binds to and promotes the mitochondrial redistribution of Drp1. The interplay promotes Drp1-regulated fission of mitochondria and, subsequently, mitoptosis [84]. An increasing number of studies indicate that disruption of mitochondria can cause promotion of autophagy. Indeed, a study reported activation of mitoptosis and the subsequent destruction of ATP was followed by the induction of autophagy to maintain the energy [107]. Another study found that clearing away abnormal mitochondria may be either be done through autophagosome formation via selective mitochondrial autophagy (mitophagy) or through the formation of mitoptotic bodies. Then they are released into the extracellular space via atypical exocytosis [108].

### 3.7. Mitophagy

Mitophagy is the process of recognizing and removing abnormal mitochondria via autophagy-regulated degradation [109]. Recent research has demonstrated that mitochondrial dynamics are essential to mitophagy. Mitochondrial fission is regulated by the GTPase dynamin-related protein 1 (Drp1). Mitochondrial fusion includes three GTPases: optic atrophy 1 (OPA1) induces inner membrane fusion and mitofusins 1 and 2 (Mfn1 and Mfn2) regulate outer membrane fusion [110]. Mitochondria are divided into depolarized and polarized mitochondria after fission. Depolarized daughter mitochondria are targeted by mitophagy, while polarized mitochondria undergo fusion [111,112]. Interestingly, accumulating evidence suggests that mitophagy undergoes extensive crosstalk with apoptosis pathways. Mitochondrial dynamics are crucial for the crosstalk between mitophagy and apoptosis. A study reported that Parkin underwent extensive crosstalk with apoptosis pathways. Mitochondrial translocation of Parkin was inhibited by pro-survival Bcl-2 proteins. It was triggered by BH3-only proteins under conditions of inhibited caspase activity [113]. Undergoing this condition for a long time, Parkin could promote apoptosis by degrading anti-apoptosis Mcl-1 [114]. Another study also found that the mitochondrial deubiquitinase USP30 opposed parkin-regulated mitophagy [115]. Knockdown of USP30 could induce the mitochondrial apoptosis pathway [116]. These findings indicated that USP30 would make mitochondria induce mitophagy rather than apoptosis. Furthermore, pre-promotion of Bnip3-mediated mitophagy by constitutively activating the Bnip3 receptor ahead of tumor necrosis factor (TNF) treatment inhibited effector caspase activation significantly [117]. It suggested that the activation of mitophagy or delayed induction of membrane permeabilization inhibited apoptosis. However, diverse feedback between individual mitophagy programs and both pro-survival and pro-death apoptosis pathways occurred at different time scales and underwent crosstalk [118].

## 4. The Relationship between Autophagy and Apoptosis in Cardiac Diseases

In physiological conditions, autophagy and apoptosis play essential roles in cardiac health and integrity. The structure and function of cardiac myocytes is closely related to autophagic flux. Cardiac myocytes retain a limited ability to enter the cell cycle again. It leads to a limited capacity for regeneration in the adult heart. As a consequence, there exists a continuous process of cell renovation. It includes removal and replacement of damaged tissue. In addition, autophagy is necessary for continual heart contraction. It is also critical for large cytoplasmic calcium transients without disturbing cardiac function [119]. During heart development, apoptosis participates in the development of the embryonic outflow tract, cardiac valves, conducting system, and coronary vasculature [120]. In pathological conditions, the interplay between autophagy and apoptosis are closely related to some cardiac diseases involving ischemic heart disease, pressure overload-induced cardiac disease and diabetic cardiomyopathy.

### 4.1. Ischemic Heart Disease

Programmed cell death of cardiac myocytes takes place following ischemia/reperfusion (I/R), leading to cardiac dysfunction. It has been proposed that I/R causes cell death via apoptosis and necrosis. Currently, it was reported that autophagy was an essential regulator of programmed cell death, either inhibiting or promoting apoptosis, or acting as a programmed cell death distinct from apoptosis. It is generally believed that promotion of autophagy is protective during myocardium ischemia. The myocardial ischemia swine models were induced by one, three, or six episodes of 90 min of left anterior descending coronary stenosis (30% decrease in baseline coronary flow) followed by reperfusion every 12 h, while the non-ischemic regions were the control. This study indicated that a chronically ischemic myocardium activated autophagy and inhibited apoptosis, which could limit the deleterious effects of chronic ischemia and protect against further ischemia [121]. It also has been shown that autophagy is activated by ischemia and reperfusion in the mouse heart *in vivo*. Under the condition of prolonged ischemia, inhibition of autophagy was accompanied by the expansion of myocardial infarction size, which suggested that the activation of autophagy protected the cardiac cells during ischemia. Moreover, it was found that ischemia induced autophagy through the AMPK-dependent signaling pathway, while reperfusion stimulated autophagy by the upregulation of Beclin-1 and BNIP3, but without AMPK activation [122,123]. In cardiac myocytes, the reduction of Beclin-1 expression by RNA interference inhibited I/R-induced autophagy, which involves enhanced cell survival [124]. The inhibition of NF-κB suppressed Beclin-1 expression and autophagy. It reduced the extent of the cardiac area at risk for ischemia [121]. It also reported that mitochondrial c-Jun N-terminal kinase (JNK) activation induced autophagy and apoptosis, aggravating myocardial I/R injury. Insulin selectively inhibited mitochondrial JNK activation, protecting cardiocytes against I/R injury. Recently, one study was aimed at investigating the effects of berberine, a natural extract from *Rhizoma coptidis*, on ischemia/reperfusion-induced excessive autophagy. Autophagy was induced both in H9c2 myocardial cells under the hypoxia/reoxygenation (H/R) condition, and in mouse hearts exposed to I/R. The results showed that berberine treatment significantly strengthened the viability of H/R-induced cells, decreased the I/R-induced myocardial infarct size, and improved the heart function. The therapeutic effect of berberine is associated with downregulating the expression of autophagy-associated proteins such as SIRT1, BNIP3, and Beclin-1, and suppressing autophagy activity. Furthermore, the levels of p-AMPK and p-mTORC2 (Ser2481) in H9c2 cardiomyocytes exposed to H/R were downregulated by berberine [125]. One study suggested that vitamin D receptor was a potential endogenous self-defensive and cardioprotective receptor protecting against myocardial I/R injury via inhibiting autophagy dysfunction–regulated cell death and apoptosis [126]. Another study indicated that sphingosylphosphorylcholine protected cardiomyocytes against ischemic apoptosis by promoting lipid raft/PTEN/Akt1/mTOR-regulated autophagy [127]. In addition, a recent work demonstrated Mst1, a crucial protein of Hippo signaling, improved the heart disorder in mice suffering myocardial infarction via suppressing autophagy. The mechanism was that Mst1 phosphorylated the Thr108 residue in the BH3 domain of Beclin1. It enhanced the interplay between Beclin1 and Bcl-2 and/or Bcl-xL, and stabilized the Beclin1 homodimer. It also suppressed the phosphatidylinositide 3-kinase activity of the Atg14L-Beclin1-Vps34 complex and subsequently inhibited autophagy. Mst1-mediated sequestration of Bcl-2 and Bcl-xL by Beclin1 activated Bax and promoted apoptosis [128]. Taken together, autophagy is activated during myocardial ischemia and further enhanced by reperfusion. Autophagy is protective during the ischemic phase, while it is harmful in reperfusion. It is supposed that activation of regular autophagy and inhibition of abnormal autophagy and apoptosis can rescue myocardial cells against death during ischemia/reperfusion.

### 4.2. Pressure Overload–Induced Cardiac Disease

Although accumulating research has paid close attention to the role of autophagy and apoptosis in pressure overload–induced cardiac disease, it is still unclear whether they play positive or negative roles in cardiac disease. A study reported that in adult mice, knockout of cardiac-specific Atg5 led to cardiac hypertrophy. It also caused left ventricular expansion one week after treatment with thoracic transverse aortic constriction (TAC). These results suggested that under baseline conditions, regular autophagy was a homeostatic mechanism for maintaining the structure and function of the heart. Autophagy activation was an adaptable reaction for preventing hemodynamic stress in heart failure [129]. Another study found that berberine could effectively attenuate cardiomyocyte apoptosis and left ventricular remodeling through an autophagy-dependent mechanism in rat cardiac hypertrophy models induced by TAC. The potential mechanism was related to inducing autophagy by the suppression of mTOR activity and its upstream p38 and extracellular signal-regulated kinase (ERK1/2) mitogen-activated protein kinase (MAPK) signaling pathways [130]. In contrast, some research suggests that autophagy has a detrimental effect on pressure overload–induced cardiac disease. A study reported that pressure overload induced by aortic banding significantly enhanced cardiac autophagy and led to heart failure. Pressure overload–induced autophagy reached the peak at 48 h. It kept rising for at least three weeks. Heterozygous disruption of Beclin-1 gene coding inhibited cardiomyocyte autophagy and alleviated pathological remodeling induced by TAC. On the contrary, Beclin-1 over-expression increased autophagy and pathological remodeling. Nevertheless, it was ambiguous if apoptosis participated in later stages of pathological remodeling [131]. Another research found that in the renal artery stenosis–induced experimental hypertensive swine model, activation of autophagy and apoptosis participated in left ventricular hypertrophy and pathological remodeling. It indicated that autophagy could portend the level of cardiac hypertrophy [132]. One study also showed that activated transcription factor 3 (ATF3) protected against pressure overload–induced heart failure. The mechanism was bound to the ATF/cAMP response element of the Beclin-1 promoter and inhibited autophagic activity by inhibition of the Beclin-1–dependent pathways [133]. In addition, the crosstalk between apoptosis and autophagy regulates proliferation and death of cells in pulmonary hypertension pathogenesis, especially in pulmonary vascular remodeling involving endothelial cells and smooth muscle cells [134].

### 4.3. Diabetic Cardiomyopathy

Diabetic cardiomyopathy has the feature of ventricular dysfunction. It turns up in many diabetic patients without coronary artery disease or hypertension. Cardiomyocytes are exposed to hyperglycemia, dyslipidemia, and oxidative stress, which can trigger both autophagy and apoptosis [135]. Accumulating research has reported that the interaction of autophagy and apoptosis is essential in diabetic cardiomyopathy. It has been found that diabetic cardiomyopathy is related to inhibition of cardiac autophagy. Induction of AMPK resumes cardiac autophagy and prevents against cardiomyopathy in diabetic mice [136]. However the exact mechanism is unclear. Digging deeper, it was reported that under the high glucose condition, AMPK activity was inhibited, JNK1-Bcl-2 signaling was suppressed, and Beclin-1 combining with Bcl-2 was promoted. On the contrary, metformin promoted AMPK and induced the JNK1-Bcl-2 pathway. Then the Beclin-1-Bcl-2 complex was destroyed. AMPK induction normalized cardiac autophagy. It also inhibited high glucose–induced apoptosis in cultured H9c2 cardiac myoblast cells. Moreover, chronic administration of metformin in diabetic mice resumed cardiac autophagy by inducing JNK1-Bcl-2 signals and separating the Beclin-1-Bcl-2 complex [137]. Another research also reported that diabetes induced apoptosis and suppressed autophagy of cardiomyocytes through inhibiting AMPK, suppressing the MAPK8/JNK1-Bcl-2 signaling pathway, and subsequently promoting the interaction between Beclin-1 and Bcl-2 [138]. Furthermore, one study showed that in streptozotocin-diabetic mice, heme oxygenase-1 prevented cardiac dysfunction via inhibiting apoptosis, inflammation, oxidative stress, and promoting autophagy [139].

## 5. Conclusions

The multiple layers of crosstalk between autophagy and apoptosis present themselves as a seamless state between survival and death in response to diverse cellular stress. Recently, emphasis has been laid on identifying and investigating of the direct protein-protein interactions between autophagic and apoptotic proteins. In this respect, an interesting question relating to the evolutionary advantage of utilizing an autophagy protein to regulate apoptosis (and *vice versa*) remains to be further studied. It is unclear whether the interactions are a true crosstalk between autophagy and apoptosis, or simply dual functional proteins mediating each process respectively. The possible mechanism for autophagy proteins regulating apoptosis is that activating the apoptotic effect of these autophagy proteins can suppress their autophagic function, leading to a decrease of pro-survival autophagy and an increase of apoptosis. Another possibility is that under severe damage conditions, specific autophagy proteins act as rheostats which can perceive the metabolic state of cells and induce apoptosis.

Furthermore, in this field the major challenge is to develop the identification of individual interactions towards a more integrative and global investigation of how they (autophagy, apoptosis or even necrosis) come together to determine the fate of cells. Special attention is also paid to the importance of the crosstalk between autophagy and apoptosis in diverse pathological progresses. A growing number of research has paid attention to the interactions of autophagy and apoptosis in the development of cancer and neurodegeneration. However, their roles in cardiovascular diseases are still under debate and have great research potential. In the future, the crosstalk between autophagy and apoptosis may be a novel and potential target for cardiovascular disease therapy.

## Figures and Tables

**Table 1:** Proteins with a dual role in autophagy and apoptosis.

Protein	Condition	Autophagy	Apoptosis	Molecular Event	References
**Bcl-2/Beclin-1**	Normal	↓	↓	Beclin-1 binding to Bcl-2	[48,49,50,51,52,53,54,55,56]
	Starvation	↑	↓	Bcl-2-Beclin-1 complex disruption; promoting autophagosome formation	[57,58,59,60,61]
	Long-term starvation	↑	↓	Phosphorylated Bcl-2 binding to Bax; preserving the mitochondrial membrane integrity; preventing pro-apoptotic proteins releasing into cytoplasm	[62]
	Extreme starvation	↓	↑	Hyper-phosphorylated Bcl-2 dissociation from Bax; caspase 3 cleavage	[62]
**ATG4**	Normal	↑	↓	Covalent attachment ATG8 to PE and delipidation of ATG8 at the lysosomal fusion stage	[63]
	Drug intervention	↓	↑	The N-terminal fragment of ATG4D cleaving and delipidating GABARAP-L1, decreasing autophagosome formation; the C-terminal fragment recruiting to mitochondrial matrix, promoting mitochondria-mediated apoptosis	[64,65]
**ATG5**	Normal	↑	↓	Promoting autophagosome formation	[66]
	Apoptotic stimuli	↓	↑	Calpains cleaving ATG5 and truncated ATG5 interacting with Bcl-X_L_ and triggering cytochrome c release and caspase activation	[67]
**ATG12**	Normal	↑	↓	Promoting autophagosome formation	[66]
	Apoptotic stimuli	↓	↑	Non-conjugated ATG12 binding to and inhibiting Mcl-1 and Bcl-2, promoting mitochondrial apoptosis; ATG12 could be directly ubiquitinated, promoting its proteasomal degradation and proteasome inhibitor-mediated apoptosis	[68,69]
**Caspase-2**	Lack of caspase-2	↑	↓	Inhibiting caspase-2-dependent apoptosis	[70,71]
**Caspase-3**	Staurosporine inducing	↓	↑	Caspase-3 cleaving Beclin-1	[72]
	IL-3 withdrawal from culture medium	↓	↑	C-terminal fragment of Beclin-1 localizing at mitochondria and sensitizing cells to apoptosis	[73]
**Caspase-6**	Apoptotic stimuli	↓	↑	Caspase-6 cleaving p62 and ATG3	[74]
	Arginine deprivation	↓	↑	Caspase-6 cleaving ATG5 and Beclin-1	[75]
**Caspase-8**	Death receptor-triggered apoptosis	↓	↑	Caspase-8 cleaving ATG3	[76]
**Caspase-9**	Interaction with Atg7	↑	↓	Caspase-9 interacting with ATG7 and promoting the ATG7-dependent formation of autophagosomal LC3-II; hindering the recruitment and processing of caspase-9 in apoptosome	[77]
	Inhibition of caspase-9	↓	↑	Blocking autophagic flux and inducing cell death	[78]
**p53**	Normal	↓	↑	In cytoplasm, p53 promoting the pro-apoptotic proteins and inhibiting Bcl-2, triggering the intrinsic apoptotic pathway; inactivating AMPK and mTOR signaling; in nucleus, p53 increasing TRAIL and Fas receptor, initiating the extrinsic apoptotic pathway; p53 activating Apaf-1 of the apoptosome	[79,80,81]
	Genotoxic stress	↑	↓	Transcriptional activation of DRAM, promoting autolysosome formation	[82]
	Nutrient deprivation	↓	↑	p53 post-transcriptionally down-regulating LC3 and controlling autophagic flux	[83]
**FLIP**	Virus infection	↓	↑	FLIP competing with LC3 for binding of ATG3 and inhibiting LC3 lipidation, suppressing autophagy	[84]

## Abbreviations

mTOR: mammalian target of rapamycin; ULK: unc-51-like kinase; Atg13: autophagy-related protein 13; FIP200: FAK-family Interacting Protein of 200 kDa; AMBRA1: activating molecule in beclin-1-regulated autophagy; PI3K: phosphatidylinositol-3-kinase; VPS15: vacuolar protein sorting 15; LAMP: lysosomal associated membrane protein; Apaf-1: apoptotic protease-activating factor-1; TNF: tumor necrosis factor; TNF-R: tumor necrosis factor-receptor; DD: death domain; FADD: fas-associated protein with death domain; TRADD: TNF-R type 1-associated death domain protein; DISC: death-inducing signaling complex; Bcl-2: B-cell lymphoma 2; JNK1: C-Jun N-terminal protein kinase 1; DAPK: death-associated protein kinase; HMGB1: high mobility group box 1; GABARAP-L1: γ-aminobutyric acid receptor-associated protein-like 1; DRAM: damage-regulated autophagy modulator; AMPK: AMP activated protein kinase; FLIP: FADD-like IL-1β-converting enzyme-inhibitory protein; TAC: transverse aortic constriction; ERK: extracellular signal-regulated kinase; MAPK: mitogen-activated protein kinase; ATF3: activating transcription factor 3; VSMC: vascular smooth muscle cells; ApoL6: apolipoprotein L6.
